# Fortification of Wheat Bread with Increasing Levels of Kudzu (*Pueraria lobata*) Root Powder: Technological, Nutritional, and Sensory Implications

**DOI:** 10.3390/foods15101824

**Published:** 2026-05-21

**Authors:** Anna Wirkijowska, Paulina Łysakowska, Piotr Zarzycki, Dorota Teterycz, Aldona Sobota

**Affiliations:** Department of Engineering and Cereals Technology, Faculty of Food Science and Biotechnology, University of Life Sciences in Lublin, Skromna 8, 20-704 Lublin, Poland; anna.wirkijowska@up.lublin.pl (A.W.); dorota.teterycz@up.edu.pl (D.T.); aldona.sobota@up.edu.pl (A.S.)

**Keywords:** antioxidant capacity, bread quality, farinograph properties, functional ingredients, starch digestibility, texture profile analysis

## Abstract

The growing interest in functional bakery products has driven research toward the incorporation of non-conventional plant materials rich in dietary fiber. In this study, the effects of partial substitution of wheat flour with ground kudzu root (*Pueraria lobata*) at levels of 3%, 6%, 9%, and 12% on dough rheology and bread quality were investigated. Farinograph analysis showed that kudzu addition slightly increased water absorption and dough development time, while significantly improving dough stability and the farinograph quality number. At the same time, a higher degree of dough softening indicated partial weakening of the gluten network at higher substitution levels. The incorporation of kudzu root significantly increased bread yield due to enhanced water retention associated with its high dietary fiber content. However, a reduction in specific volume was observed at the highest substitution level (12%), indicating limitations in gas retention capacity. Crumb structure analysis revealed a shift toward a finer and more homogeneous pore distribution with increasing kudzu content, accompanied by a reduction in large pores. These structural changes were reflected in texture profile analysis, where increased hardness and chewiness were observed, particularly at higher substitution levels, while cohesiveness and springiness were only slightly affected. Partial substitution with kudzu root powder also resulted in a significant increase in total phenolic content, flavonoid content, and antioxidant potential of the breads, with the highest values observed in samples containing 12% kudzu root powder. In addition, breads enriched with kudzu root showed reduced digestible starch content compared with the control sample. Despite these modifications, breads enriched with up to 9% kudzu root maintained acceptable technological quality, balancing improved water retention with moderate changes in structure and texture. The results demonstrate that kudzu root can be used as a functional ingredient in wheat bread, contributing to increased dietary fiber content while maintaining satisfactory processing and quality characteristics.

## 1. Introduction

Wheat bread is one of the most widely consumed cereal products worldwide and constitutes an important source of energy, carbohydrates, B-group vitamins, and minerals in the daily diet [1]. However, modern bread production is largely based on refined wheat flour, the high degree of processing of which leads to a significant loss of dietary fiber, minerals, and phenolic compounds naturally present in the bran and germ of the grain [2]. As a result, many commercial bakery products made from wheat flour are characterized by reduced nutritional value compared with whole-grain products [3,4].

In recent years, growing awareness of the role of diet in the prevention of diet-related diseases has contributed to increased interest in developing bakery products with improved nutritional composition while maintaining appropriate technological and sensory properties [5]. One strategy for improving the nutritional value of bread is the partial substitution of wheat flour with plant-derived ingredients such as legumes, tubers, roots, or pseudocereals. The incorporation of such additives affects the technological properties of dough primarily through dilution of the gluten network, alteration of water absorption capacity, and modification of starch gelatinization behavior [6,7]. Dietary fiber present in plant additives may compete with gluten proteins for water or create physical barriers that limit the formation of the gluten structure (the so-called steric hindrance effect). Moreover, phenolic compounds present in these materials may interact with gluten proteins through hydrogen bonding and hydrophobic interactions [8,9]. Consequently, the use of plant-based additives may lead to changes in crumb structure and bread volume; however, it simultaneously contributes to an improvement in nutritional value [5,10].

One plant raw material with potential application in the production of functional foods is kudzu (*Pueraria lobata* (Willd.) Ohwi, Fabaceae), a perennial climbing plant naturally occurring in China, Japan, and Korea. For over two thousand years, this plant has been used in East Asia both as a food ingredient and as a medicinal resource. Dried kudzu roots (*Radix Puerariae Lobatae*) are listed in the Chinese Pharmacopoeia and are used, among others, as ingredients in beverages, desserts, and functional foods [11,12]. Interest in this raw material arises primarily from its rich chemical composition and the presence of numerous bioactive compounds. Kudzu root contains considerable amounts of starch, dietary fiber, protein, and phenolic compounds. On a dry matter basis, starch content may reach approximately 40–50%, whereas the content of insoluble and soluble dietary fiber is approximately 10 g/100 g and 3.8 g/100 g, respectively [11,13]. Kudzu starch is characterized by crystalline polymorphism of type A or C and high final viscosity, which may be of technological significance in food processing applications [14,15].

An important component of the chemical composition of kudzu is the isoflavonoid fraction. More than 50 individual compounds belonging to this group have been identified in the root, among which puerarin, daidzin, and daidzein are quantitatively the most significant [16]. Puerarin constitutes a characteristic chemotaxonomic marker of kudzu and has been associated with a broad spectrum of biological activities, including antioxidant and anti-inflammatory properties [17,18,19]. The presence of phenolic compounds may also influence the technological properties of food products as well as their antioxidant activity [20,21]. Previous studies on kudzu have mainly focused on the physicochemical properties of isolated kudzu starch and its use as a thickening and gel-stabilizing agent or as an ingredient in cereal-based products such as noodles [14,15,21,22]. However, data regarding the use of whole kudzu root powder as an additive in wheat bread remains limited.

Therefore, the aim of the present study was to determine the effect of partial substitution of wheat flour with commercial whole kudzu root powder (*Pueraria lobata*) at levels of 3, 6, 9, and 12% (*w*/*w*) on the technological properties of wheat bread as well as on its chemical composition and nutritional value.

## 2. Materials and Methods

### 2.1. Materials

The wheat flour (type 750) used as the primary raw material in this study was purchased from a local supermarket in Lublin, Poland. The flour exhibited the following characteristics: moisture content of 12.48% ± 0.45%, ash content of 0.73% (d.b.), wet gluten content of 26.9% ± 0.5%, dry gluten content of 9.53% ± 0.2%, gluten water absorption capacity of 17.3% ± 0.3%, gluten index of 97.0 ± 1.0, falling number of 289 ± 10 s, and particle size below 0.2 mm for 99% of the sample. The kudzu root powder (Lat. *Pueraria lobata*) used in this study was obtained from the commercial supplier Lymeherbs (Chorzów, Poland). The material was provided in the form of a finely milled powder derived from dried kudzu root. According to the manufacturer’s specification, the product contained 100% powdered kudzu root without additives or preservatives (particle size below 0.150 mm for 99% of the sample). The powder was light beige in color and exhibited a characteristic mild herbal odor. Prior to use, the material was stored in airtight containers at room temperature in a dry and dark environment to prevent moisture uptake and degradation.

### 2.2. Bread Preparation

Wheat bread samples were prepared using the straight dough method, as described by Zarzycki et al. [23] and Wirkijowska et al. [24], with minor modifications to accommodate the partial substitution of wheat flour with kudzu root powder. Five bread variants were produced: a control sample (CON) contained only wheat flour, water, yeast, and salt, and four experimental variants (BKRF3, BKRF6, BKRF9, BKRF12) in which 3%, 6%, 9%, and 12% of wheat flour was replaced with kudzu root powder, respectively. Each formulation was prepared in two separate baking trials, with three loaves in each trial, yielding a total of six loaves per variant. A randomized design was used to reduce the impact of possible batch effects and to ensure statistical reliability.

The basic recipe for each bread variant consisted of 600 g of flour (or kudzu root powder mixture), 18 g of compressed fresh yeast, 9 g of sodium chloride, and the quantity of water determined individually for each sample by farinographic analysis in accordance with AACC Method 54-21.01 [25]. All dry ingredients were thoroughly combined prior to adding water and yeast. The dough was mixed for 3 min at low speed (level 1) using a laboratory spiral mixer (BEAR Varimixer Teddy 5 L, Varimixer A/S, Brøndby, Denmark), and then at high speed until a homogeneous dough was obtained and full gluten development was achieved. The mixed dough was placed in a proofing chamber (Tefi Klima Pro 100, Debag, Bautzen, Germany) and fermented at 30 ± 1 °C and 85% ± 2% relative humidity for 90 min, with an intermediate punching step applied after 60 min (30 s at low speed). Following this step, the dough was portioned into 290 ± 5 g pieces, molded, and placed in baking tins (18 × 7.5 × 7.0 cm). The shaped dough was then subjected to final proofing at 30 ± 1 °C and controlled humidity (75–85%) for 30 min. Baking was carried out in a deck oven (Helios Pro 100, Debag, Germany) at 230 °C for 35 min. After removal from the pans, the loaves were cooled at room temperature (22 ± 1 °C, 50% RH) for 1 h, weighed, packaged in polyethylene bags, and stored under ambient conditions until analysis.

### 2.3. Farinograph Properties of Dough

The rheological properties of dough prepared from wheat flour partially replaced with kudzu root (0–12%) were evaluated using farinograph analysis, following the methodology described by Wirkijowska et al. [26]. The parameters assessed included water absorption (WA), dough development time (DDT), stability time (ST), dough softening (DS), and farinograph quality number (FQN). Measurements were carried out using a Farinograph-TS (Brabender, Duisburg, Germany) in accordance with AACC Method 54-21 [25], applying the constant flour weight procedure (300 g, 14% moisture basis). Each sample was analyzed in triplicate.

### 2.4. Bread Evaluation

Bread yield (BY, % Equation (1)), baking loss (BL, %, Equation (2)), specific volume (SV, cm^3^ g^−1^), and moisture content after 24 h and 72 h of storage were determined following the procedures reported by Zarzycki et al. [23], Wirkijowska et al. [24,26]. Bread yield was calculated as the ratio of the mass of the cooled loaf (1 h after baking) to the total mass of flour used in the recipe, expressed as a percentage (Equation (1)):

BY (%) = (*m*_*bread*_/*m*_*flour*_) × 100 (1)

Baking loss was calculated from the difference in dough weight before baking (*m_dough_*) and bread weight after cooling (*m_bread_*), expressed as a percentage of the initial dough weight (Equation (2)):

BL (%) = [(*m*_*dough*_ − *m*_*bread*_)/*m*_*dought*_] × 100 (2)

Bread volume was measured using the rapeseed displacement method according to AACC Method 10-05.01 [25]. Specific volume was calculated as the ratio of loaf volume (cm^3^) to loaf mass (g). Crumb moisture content was determined at two time points following baking: 24 h and 72 h. Samples were taken from the central part of the loaf, and moisture was measured by drying at 105 °C to constant mass, according to AACC Method 44-15.02 [25]. The measurements were performed in triplicate (*n* = 3) for each bread variant and storage time point.

### 2.5. Porosity of Bread

The porosity of bread crumbs was evaluated as described previously [26], using a VHX-7000 digital microscope (Keyence Corporation, Osaka, Japan) with dedicated analytical software. Images were captured from the central part of each bread slice, covering a 4 × 4 cm area, at 20× magnification, a 0° observation angle, and with ring light illumination. The automatic grain measurement function based on brightness extraction was applied, and the hole-filling option was used to enhance the accuracy of pore identification. Only pores with a minimum diameter of 0.01 mm were included in the analysis. Each bread sample was analyzed in triplicate, and both the number and surface area of pores were recorded. Individual pore size data, expressed in mm^2^, were assigned to five size classes: 0.01–0.04 mm^2^, 0.05–0.09 mm^2^, 0.1–0.9 mm^2^, 1–4 mm^2^, and >4 mm^2^. For each class, the pore count and cumulative surface area were determined. The results were additionally expressed as the percentage share of the total pore count and total pore surface area.

### 2.6. Texture Profile Analysis (TPA) of Bread

Bread crumb texture was evaluated using the Texture Profile Analysis (TPA) method described by Wirkijowska et al. [26]. After baking, the loaves were sliced into 20 mm thick slices, and the crust was carefully removed. Cuboidal crumb samples (30 × 30 × 20 mm) were prepared for analysis. The double compression test was performed using a Brookfield AMETEK CTX Texture Analyzer (AMETEK Brookfield Inc., Middleboro, MA, USA) equipped with a flat cylindrical probe (50 mm diameter). Samples were compressed to 50% of their original height at a crosshead speed of 1 mm/s, with a trigger force of 0.98 N and a maximum load of 500 N. The following TPA parameters were determined using Texture Pro software (v.1.0.19): hardness (N), cohesiveness (−), springiness (−), and chewiness (N). Measurements were carried out 24 and 72 h after baking. For each formulation, eight independent replicates were analyzed.

### 2.7. Evaluation of Color Parameters of Bread Crumb

The color of bread crumb was determined in the CIE L*a*b* color space using a Chroma Meter CR-5 spectrophotometer (Konica Minolta, Sakai, Osaka, Japan), following the procedure described by Wirkijowska et al. [26]. The parameters measured included L* (lightness), a* (redness/greenness), and b* (yellowness/blueness). Prior to analysis, the instrument was calibrated using standard black and white reference plates. Measurements were performed under a D65 illuminant, with a 10° standard observer and an 8 mm measurement aperture. Each sample was analyzed in 12 replicates. Based on the obtained L*, a*, and b* values, the total color difference (ΔE*; Equation (3)) relative to the control sample was calculated. Additionally, the whiteness index (WI; Equation (4)), yellowness index (YI; Equation (5)), and browning index (BI; Equation (6)) were determined according to equations reported in the literature.


(3) $$∆E^{*}=\sqrt{(L_{c}^{*}-L_{i}^{*}{)}^{2}+(a_{c}^{*}-a_{i}^{*}{)}^{2}+(b_{c}^{*}-b_{i}^{*}{)}^{2}}$$

(4) $$WI=100-\sqrt{\left((100-L^{*}{)}^{2}+a^{2}+b^{2}\right)}$$

(5) $$YI=142.83\cdot \frac{b^{*}}{L^{*}}$$

(6) $$BI=\frac{\left[\left(X-0.31\right)\cdot 100\right]}{0.17};where\;X=\frac{a^{*}+1.75\cdot L^{*}}{5.645\cdot L^{*}+a^{*}-3.012\cdot b^{*}}$$

### 2.8. Sensory Evaluation of Bread

The sensory evaluation of bread was carried out by a twelve-member panel of experts selected based on their experience in food product assessment, regular consumption of bakery products, and absence of gluten allergies. The panelists were trained in accordance with ISO 8586:2012 [27] and familiarized with the principles of sensory analysis using a five-point scale. The evaluation was performed under controlled laboratory conditions with standardized lighting, temperature, and humidity, in accordance with ISO 8589:2007 [28]. The study received approval from the Bioethics Committee of the University of Life Sciences in Lublin, Faculty of Food Sciences and Biotechnology (Resolution No. UKE/09/2023). The following sensory attributes were assessed: appearance, crumb color, elasticity and porosity, smell, taste, and overall acceptability. Bread samples were sliced into 1 cm-thick pieces, coded, and presented to the panelists in a random order. Between evaluations, panelists cleansed their palate with water, while coffee was used only as an olfactory neutralizer during aroma assessment. The results were recorded on evaluation sheets using a five-point scale, where 5 indicated the highest quality and 1 the lowest.

### 2.9. Chemical Composition of Raw Materials and Bread

The proximate composition of raw materials and bread samples obtained from different formulations was determined using standardized AACC and AOAC methods [25,29]. Moisture and ash contents were measured according to AACC Methods 44-15A and 08-01, respectively. Protein content was determined using the Kjeldahl method (AACC 46-08), applying a nitrogen-to-protein conversion factor of 5.7. Fat content was analyzed following AACC Method 30-26. Total dietary fiber was quantified using AOAC Methods 991.43 and 985.29, supported by corresponding AACC procedures (32-07, 32-21, and 32-05). The content of available carbohydrates was calculated by difference, subtracting the sum of moisture, ash, protein, fat, and total dietary fiber from the total sample weight. The energy value of the samples was estimated using Atwater conversion factors (4 kcal/g for protein and carbohydrates, 9 kcal/g for fat, and 2 kcal/g for dietary fiber). All analyses were performed in triplicate.

### 2.10. Determination of Total Phenolic Content, Total Flavonoid Content, and Antioxidant Capacity

The extraction procedure and determination of total phenolic content (TPC), total flavonoid content (TFC), and antioxidant capacity (DPPH and ABTS assays) were carried out according to previously described methods with slight modifications [30,31,32,33,34]. Briefly, wheat flour, kudzu root and bread samples were extracted using 70% (*v*/*v*) ethanol at 40 °C. The obtained extracts were subsequently used for spectrophotometric determination of TPC using the Folin–Ciocalteu reagent, TFC using the aluminum chloride method, and antioxidant capacity based on DPPH and ABTS radical scavenging assays. Absorbance measurements were performed with a Thermo Spectronic Helios Epsilon spectrophotometer (Thermo Electron, Waltham, MA, USA). Results were expressed as gallic acid equivalents (GAE), quercetin equivalents (QE), and Trolox equivalents (TE) per gram of product dry matter. All determinations were conducted in triplicate.

### 2.11. Determination of Digestible Starch

Digestible starch was determined as total digestible starch (TDS) using the Digestible and Resistant Starch Assay Kit (K-DSTRS, Megazyme, Neogen Corporation, Lansing, MI, USA), according to the manufacturer’s protocol. In the K-DSTRS procedure, TDS is defined as the starch fraction hydrolysed during 240 min of enzymatic incubation with pancreatic α-amylase and amyloglucosidase. The K-DSTRS protocol also enables the determination of rapidly digestible starch (RDS), slowly digestible starch (SDS), and resistant starch (RS); however, only TDS results are presented in the present study.

Briefly, samples were incubated with pancreatic α-amylase and amyloglucosidase in maleate buffer at 37 °C for 4 h. After incubation, an aliquot of the reaction mixture was collected, and the enzymatic reaction was terminated with 50 mM acetic acid. The solution was centrifuged, and the released glucose was quantified colorimetrically after reaction with the GOPOD reagent (glucose oxidase/peroxidase and 4-aminoantipyrine). Absorbance was measured at 510 nm using a spectrophotometer (Thermo Electron, Waltham, MA, USA). The amount of TDS was calculated from the measured glucose concentration using the conversion factor 162/180 and expressed as g per 100 g of sample dry matter (g/100 g d.m.). The results were expressed as mean ± standard deviation. Each measurement was performed in duplicate. Calibration curves for glucose determination showed high linearity (R^2^ ≥ 0.989).

### 2.12. Statistical Analysis 

All analytical results were expressed as mean values accompanied by their corresponding standard deviations. Statistical analysis was performed using one-way and two-way analysis of variance (ANOVA), depending on the nature of the evaluated data. One-way ANOVA was used to assess differences among formulations within a given storage period. For texture profile analysis (TPA), a two-way ANOVA was applied to evaluate the effects of two fixed factors, namely kudzu root addition level and storage time, as well as their interaction. When significant effects were detected, Tukey’s multiple comparison test was used to identify differences between mean values at a significance level of *p* ≤ 0.05. All statistical analyses were carried out using Statistica software (version 13.3, StatSoft Inc., Tulsa, OK, USA). Each determination was conducted in at least triplicate to ensure the reliability and repeatability of the results. 

## 3. Results and Discussion

### 3.1. Farinograph Properties of Dough

The farinographic characteristics of wheat dough supplemented with kudzu root are presented in Table 1. The results indicate that the addition of kudzu root (3–12%) modified dough rheology, although not all changes were statistically significant. Water absorption (WA) showed a gradual increase from 59% in the control to 63% in the sample with 12% kudzu root; however, these differences were not statistically significant (*p* > 0.05). This trend may be attributed to the high dietary fiber content of kudzu root, particularly insoluble fiber (36.6%), which enhances water-binding capacity due to the presence of numerous hydroxyl groups capable of forming hydrogen bonds with water. Similar relationships between fiber content and increased water absorption have been widely reported [23,35,36].

A slight increase in dough development time (DDT) was also observed (from 5.9 to 6.5 min), although these changes were not statistically significant (*p* > 0.05). The extension of DDT may suggest a delay in gluten network formation, likely due to gluten dilution and the presence of non-gluten components such as fiber and low-protein material in kudzu root (6.6% vs. 13.4% in wheat flour), which can interfere with protein hydration and network development.

In contrast, dough stability time (ST) increased significantly (*p* ≤ 0.05), from 5.9 min in the control to 10.8 min at 12% substitution. This indicates increased apparent resistance to mechanical mixing; however, in fiber-enriched systems, such changes may result from enhanced water binding and increased dough viscosity rather than from a true strengthening of the gluten network. This observation is further supported by the increase in the Farinograph Quality Number (FQN), which rose from 97 to 132 with increasing kudzu content, suggesting altered dough mixing characteristics rather than an unequivocal improvement in dough quality.

However, dough softening (DS) increased markedly and significantly (from 47 to 105 FU), indicating a greater susceptibility of the dough to weakening during prolonged mixing. The pronounced increase in this parameter suggests that the gluten network became less cohesive and more prone to breakdown, despite the higher stability values. Similar effects have been reported for fiber-rich by-products, where the balance between reinforcement and weakening of the gluten matrix depends on the level and type of fiber incorporation [23].

In summary, the incorporation of kudzu root resulted in increased water absorption, extended dough development, and significant changes in dough stability and Farinograph Quality Number (FQN). These effects likely reflect enhanced water immobilization and increased viscosity rather than a direct improvement in gluten strength. At the same time, the substantial increase in dough softening indicates a partial weakening of the gluten network, highlighting the complex role of fiber-rich ingredients in shaping dough rheology. These rheological modifications may influence subsequent bread characteristics, particularly water retention, loaf volume, and crumb structure.

### 3.2. Bread Evaluation

The physical properties of wheat breads with partial substitution of wheat flour by kudzu root powder are presented in Table 2. The addition of kudzu root powder significantly affected bread yield and specific volume, while baking loss and crumb moisture showed more moderate responses.

Bread yield increased progressively and significantly (*p* ≤ 0.05) with increasing levels of kudzu root powder substitution, ranging from 129.5% in the control to 148.8% in BKRF12. This effect is consistent with the higher water absorption observed in farinograph analysis (Table 1) and can be attributed to the high dietary fiber content of kudzu root (TDF ≈ 41.7%d.b.), particularly its insoluble fraction (IDF = 36.6%), which effectively binds and retains water during processing. Similar behavior has been reported for other fiber-enriched bread systems [23].

The baking loss (BL) values for the analyzed bread samples ranged from 10.5% to 11.6% and did not exhibit a clear linear trend with increasing substitution level (Table 2). The obtained values fall within the range typically reported for wheat bread enriched with dietary fiber-rich materials [23,24]. The absence of a consistent trend in BL may be attributed to the combined effects of the dietary fiber and starch properties of kudzu root. The high total dietary fiber content (TDF ≈ 41.7% d.m.) contributes to increased water binding, as reflected in the higher water absorption values observed in farinograph analysis (Table 1). Although greater water retention would be expected to reduce baking loss, this effect may be partially offset by the characteristics of kudzu starch. Its relatively high gelatinization temperature may delay or limit starch gelatinization during baking, resulting in a fraction of unbound water that is more susceptible to evaporation [37,38]. Additionally, gluten dilution caused by the incorporation of a non-gluten component may further influence water retention and gas-holding capacity. Overall, the BL values observed for all variants can be considered technologically acceptable.

The specific volume of the loaves remained comparable to the control up to 9% substitution, while a significant decrease was observed at the highest level (12%). This reduction is associated with weakening of the gluten network, as indicated by increased dough softening (Table 1). The presence of non-gluten components derived from kudzu root, including dietary fiber and starch, likely interfered with gluten development and gas cell stabilization, thereby limiting loaf expansion [6]. Similar trends have been reported for bread enriched with fiber-rich plant materials [24,26,38].

Crumb moisture content measured after 24 h did not differ significantly among samples and remained within a narrow range (44.5–44.7%). During storage, however, the control sample showed a significant decrease in moisture, whereas kudzu-enriched breads maintained relatively stable moisture levels. This behavior suggests that the dietary fiber present in kudzu root limited water migration within the crumb, thereby enhancing moisture retention during storage. Such an effect is consistent with the well-documented role of dietary fiber in reducing crumb drying and slowing staling in fiber-enriched bread systems [26].

### 3.3. Porosity of Bread Samples

**Figure 1:**
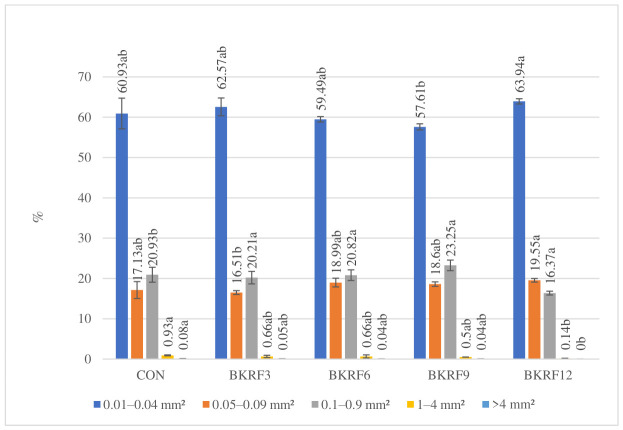
Percentage distribution of crumb pores by number across defined size ranges. Different lowercase letters above the bars indicate statistically significant differences between samples (*n* = 4; Tukey’s test, *p* ≤ 0.05).

**Figure 2:**
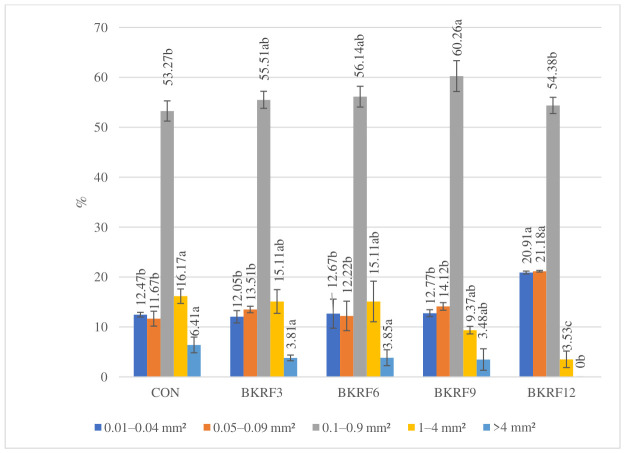
Percentage distribution of crumb pores by surface area across defined size ranges. Different lowercase letters above the bars indicate statistically significant differences between samples (*n* = 4; Tukey’s test, *p* ≤ 0.05).

The porosity analysis of bread crumbs revealed significant differences in pore size distribution among the samples analyzed. In terms of pore count (Figure 1), the smallest pore class (0.01–0.04 mm^2^) was dominant in all samples, accounting for 57.61–63.94% of the total number of pores, regardless of the substitution level. However, when expressed as a percentage of total pore surface area (Figure 2), the dominant fraction shifted to the 0.1–0.9 mm^2^ class, contributing 53.27–60.26% of the total pore area. This indicates that, although small pores were numerically predominant, medium-sized pores contributed most to the crumb structure due to their larger size.

In the control sample (CON), the pore structure was relatively heterogeneous. While the 0.1–0.9 mm^2^ fraction dominated (53.27%), larger pores (1–4 mm^2^ and >4 mm^2^) accounted for a considerable share of the total pore surface area. This distribution is typical of well-developed wheat bread, in which a continuous and elastic gluten network enables gas cell expansion and partial coalescence during fermentation and baking [39].

With increasing levels of kudzu root powder, a progressive shift toward a finer and more uniform crumb structure was observed. The contribution of large pores (1–4 mm^2^) decreased significantly (*p* ≤ 0.05), while pores exceeding 4 mm^2^ were no longer present in samples with the highest substitution level. At the same time, the relative share of small pores increased, particularly in BKRF12. Notably, the proportion of medium-sized pores (0.1–0.9 mm^2^) reached its highest value in BKRF9, suggesting that this level of substitution may provide a favorable balance between maintaining gluten structure and incorporating dietary fiber.

These structural changes can be attributed to the high content of insoluble dietary fiber in kudzu root, which disrupts the continuity of the gluten network and limits gas cell expansion during fermentation. Additionally, the dilution of gluten-forming proteins reduces the ability of the dough to retain gas, leading to the formation of a denser crumb structure [39,40]. Similar shifts toward finer and more homogeneous pore structures have been reported for wheat bread enriched with plant materials [26].

The observed changes in crumb porosity are consistent with the reduction in specific volume (Table 2) and are reflected in the textural properties of the bread, particularly increased hardness and chewiness, as discussed in the following section.

### 3.4. Texture Profile Analysis (TPA) of Bread

Textural parameters are crucial for assessing the sensory characteristics and storage stability of bread. Among the key attributes, hardness, springiness, cohesiveness, and chewiness are commonly used to describe crumb structure and consumer perception [24,36,41,42]. The results of the texture profile analysis (TPA) of bread enriched with kudzu root are presented in Table 3. To better evaluate the effects of both formulation and storage, the TPA data were additionally subjected to two-way analysis of variance (ANOVA), with kudzu root level and storage time as fixed factors. The analysis revealed that storage time significantly affected hardness, cohesiveness, and springiness (*p* < 0.001), while its effect on chewiness was marginally non-significant (*p* = 0.053). The level of kudzu root addition significantly influenced hardness, springiness, and chewiness (*p* < 0.01), but had no significant effect on cohesiveness (*p* = 0.691). A significant interaction between formulation and storage time was observed only for cohesiveness (*p* < 0.001), indicating that changes in this parameter during storage depended on the level of kudzu root addition.

Crumb hardness, considered a primary indicator of bread freshness, increased with both storage time and the level of kudzu addition. After 24 h, no significant differences were observed between the control and samples containing up to 6% kudzu root; however, a significant increase was noted at higher substitution levels, with BKRF12 reaching 15.5 N compared to 9.6 N for the control. After 72 h, hardness increased in all samples, reflecting typical staling behavior. The increase in hardness at higher substitution levels may be attributed to the denser crumb structure and reduced specific volume (Table 2). These observations were confirmed by two-way ANOVA, which demonstrated significant main effects of both storage time and formulation (*p* < 0.001), while the interaction between these factors was not statistically significant (*p* = 0.078). This indicates that the increase in hardness during storage followed a similar pattern across all formulations. A similar increase in hardness after the incorporation of kudzu-derived ingredients was reported by Wang et al. [43], who attributed this effect to the high fiber content and disruption of the gluten-starch matrix. Comparable relationships between increased fiber content, reduced loaf volume, and higher crumb hardness have also been widely reported for other fiber-rich additives such as fruit pomace [41,42].

Cohesiveness showed only minor changes with increasing levels of kudzu root and during storage. Although a slight decreasing trend was observed, differences were generally not statistically significant, indicating that the internal bonding of the crumb structure remained relatively stable, which is consistent with findings reported for other fiber-rich ingredients [42,44]. The two-way ANOVA confirmed that formulation had no significant effect on cohesiveness (*p* = 0.691), whereas storage time significantly affected this parameter (*p* < 0.001). Moreover, a significant interaction between formulation and storage time (*p* < 0.001) was detected, suggesting that the magnitude and direction of cohesiveness changes during storage varied depending on the level of kudzu root addition. In contrast, Wang et al. [43] reported a significant decrease in cohesiveness in steamed bread enriched with kudzu, which they explained by gluten dilution and weakening of the starch-protein matrix.

Springiness decreased slightly with increasing kudzu content and during storage, reflecting reduced elasticity and typical staling processes associated with starch retrogradation. However, the magnitude of these changes was limited, suggesting that kudzu root had only a minor effect on crumb resilience. Despite the relatively small numerical differences, two-way ANOVA indicated significant effects of both storage time and formulation on springiness (*p* < 0.001), whereas their interaction was not significant (*p* = 0.126). This suggests that the reduction in crumb elasticity during storage occurred consistently across all formulations. This is in partial agreement with Wang et al. [43], who observed a more pronounced reduction in springiness, likely related to a greater disruption of the gluten network and a coarser crumb structure.

Chewiness increased significantly with higher levels of kudzu root addition, particularly at 12%, where values reached 79 N (24 h) and 81 N (72 h), compared to 62 N and 61 N for the control, respectively. This parameter, which combines hardness, cohesiveness, and springiness, reflects the increased energy required to masticate the bread. The observed increase in chewiness is consistent with the higher hardness and lower specific volume of the enriched bread (Table 2), indicating a denser and more compact crumb structure. Two-way ANOVA confirmed a significant effect of formulation on chewiness (*p* = 0.002), while the effect of storage time was close to, but did not reach, statistical significance (*p* = 0.053). The interaction between formulation and storage time was also not significant (*p* = 0.090), indicating that the increase in chewiness was primarily associated with the level of kudzu root addition. While Wang et al. [43] reported a decrease in chewiness, this discrepancy may be attributed to differences in product formulation (steamed bread vs. baked bread) and the extent of structural weakening induced by the added ingredients.

Overall, the incorporation of kudzu root resulted in increased hardness and chewiness, particularly at higher substitution levels, while cohesiveness and springiness were only slightly affected. The changes observed during storage confirm typical staling behavior. The two-way ANOVA demonstrated that both storage time and formulation significantly influenced several textural attributes, although their combined effect was significant only for cohesiveness. These results indicate that kudzu root influences crumb texture primarily through its impact on dough structure and gas retention, leading to a firmer and less elastic crumb at higher inclusion levels.

### 3.5. Evaluation of Color Parameters of Bread Crumb

**Figure 3:**
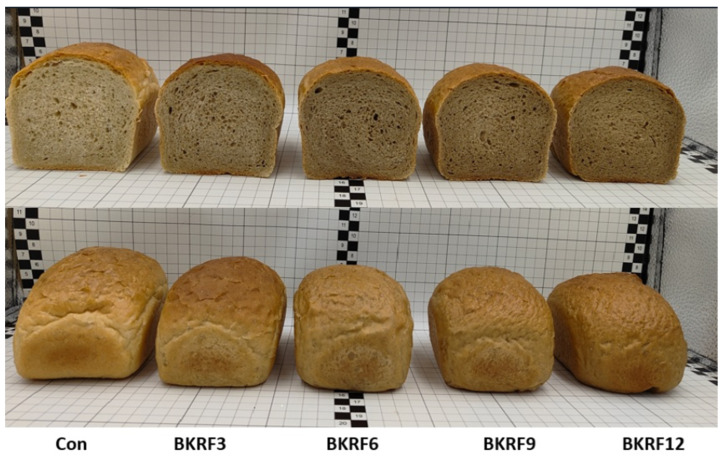
Cross-sections and surface of bread with progressive addition of kudzu root powder (0–12%).

The color parameters of bread crumbs are presented in Table 4. The incorporation of kudzu root significantly affected crumb color, resulting in a progressive darkening and a shift toward warmer tones. The L* value (lightness) decreased significantly (*p* ≤ 0.05) with increasing levels of substitution, from 55 in the control to 44 in the sample with 12% kudzu root, indicating a darker crumb. At the same time, both a* and b* values increased, reflecting a shift toward red and yellow hues, respectively. Consequently, the total color difference (ΔE*) increased with the level of enrichment, confirming that even low levels of kudzu addition led to visually perceptible changes in crumb color. These differences in crumb color are also visually evident in Figure 3, which shows a gradual darkening of the bread crumb with increasing levels of kudzu root addition. The enriched samples exhibit a slightly more yellow-brown tone compared with the lighter crumb of the control bread.

These changes can be explained by the chemical composition of kudzu root. Kudzu root is rich in flavonoids and related phenolic compounds, such as puerarin, daidzein, luteolin, and xanthones, which are typically associated with yellow hues [45,46]. The increase in b* values observed in this study is consistent with the presence of these compounds. Additionally, the rise in a* values and the simultaneous decrease in the whiteness index (WI) and increase in browning index (BI) suggest the formation of brown-colored compounds during baking. This may be attributed to the presence of condensed tannins and other polyphenols in kudzu root, which can undergo thermal transformations and participate in non-enzymatic browning reactions. Therefore, the observed color changes are most likely the result of a combination of intrinsic pigment contribution (mainly yellow flavonoids) and intensified Maillard and caramelization reactions during thermal processing.

Overall, the addition of kudzu root led to darker, more intensely colored bread crumbs with increased red and yellow tones, which may influence consumer perception, particularly at higher substitution levels.

### 3.6. Sensory Evaluation

The results of the sensory evaluation of bread with partial wheat flour substitution by kudzu root powder are presented in Table 5. The incorporation of kudzu root powder resulted in a gradual decline in scores across most of the evaluated attributes, with the magnitude of the decrease being proportional to the substitution level. Among the assessed characteristics, elasticity and porosity were the most resistant to change, showing no significant differences (*p* > 0.05) between any of the enriched samples and the control. In contrast, taste was the most sensitive attribute, with the highest substitution level receiving a score more than 50% lower than the control.

The pronounced reduction in taste acceptability at higher substitution levels can be attributed to the characteristic flavor profile of *Pueraria lobata* root, which contains bitter-tasting isoflavones, particularly puerarin and daidzein, as well as saponins that may contribute astringent and earthy notes [19]. These compounds, while contributing to the bioactive composition of kudzu root powder, may negatively influence the sensory acceptance of enriched food products when present at elevated concentrations. A comparable dose-dependent decline in taste and overall acceptability has been reported for bread fortified with other phenolic-rich plant materials, such as grape pomace powder [47], green leaf flours [48], and fermented chickpea flour [49]. It is noteworthy that the overall acceptability of bread enriched with up to 6% kudzu root powder did not differ significantly from the control (*p* > 0.05), indicating that moderate substitution levels may provide a favorable balance between nutritional enhancement and sensory acceptance. However, higher substitution levels negatively affected sensory acceptance, particularly taste, which may limit consumer acceptance at elevated enrichment levels.

### 3.7. Chemical Composition of Raw Materials and Bread Samples

The chemical composition of the raw materials and breads containing increasing amounts of powdered kudzu root is presented in Table 6. Kudzu root exhibited a distinctly different chemical profile compared to wheat flour, which was directly reflected in the composition of the baked breads. The ash content in kudzu root was 7.5 times higher than that in wheat flour, which is consistent with literature data indicating a rich mineral fraction in this raw material, including potassium, calcium, phosphorus, and selenium [46]. The protein content in kudzu root (6.6% d.b.) was lower than in wheat flour (13.4% d.b.), although kudzu protein is characterized by a favorable amino acid profile dominated by leucine and aspartic acid, as well as nutritional properties similar to those of animal protein [12,46].

Particularly noteworthy is the high dietary fiber content in kudzu root (41.7% d.b.), indicating the high potential of this raw material in enhancing the health benefits of bread. Kudzu root was also characterized by a significantly lower content of carbohydrates (45.4% d.b.) and energy value (278.2 kcal/100 g) compared to wheat flour (80.2% d.b. and 340.2 kcal/100 g, respectively), which results from the presence of dietary fiber that is not subject to enzymatic digestion [46].

Differences in the chemical composition of the raw materials were clearly reflected in the composition of the baked breads. Ash content increased significantly with increasing kudzu root content in the recipe (*p* ≤ 0.05), reaching 3.05% d.b. in the BKRF12 sample compared to 2.51% d.b. in the control sample. Protein content showed a slight downward trend; a statistically significant difference was observed only between the BKRF12 sample (12.6% d.b.) and the CON (13.8% d.b.), which is a consequence of the dilution of the protein fraction of wheat flour by a raw material with a lower protein content. Fat content increased slightly (0.44–0.50% d.b.), but due to the very low absolute values, this change is of marginal technological significance.

The most pronounced effect of wheat flour substitution with kudzu root powder was recorded for dietary fiber. TDF content increased significantly at every substitution level (*p* ≤ 0.05), from 6.2% d.b. in the control sample to 10.3% d.b. in BKRF12, corresponding to a 66% increase. This rise was predominantly driven by an increase in the insoluble dietary fiber fraction (IDF), which more than doubled, from 2.5% d.b. (CON) to 6.7% d.b. (BKRF12). The SDF content did not differ significantly among the bread variants (*p* > 0.05). The digestible carbohydrate content decreased systematically—from 77.1% d.b. (CON) to 73.6% d.b. (BKRF12)—and the energy value decreased statistically significantly at each successive substitution level, from 210.4 kcal/100 g (CON) to 204.5 kcal/100 g (BKRF12).

### 3.8. Total Phenolic Content, Total Flavonoid Content, and Antioxidant Capacity

The total content of phenolic compounds (TPC), including flavonoids (TFC), as well as the antioxidant capacity of raw materials and bread samples are presented in Table 7. Powdered kudzu root was characterized by more than a 220-fold higher TPC and a significantly higher TFC compared with wheat flour, in which flavonoids were not detected. These results confirm that the root of *Pueraria lobata* represents a rich source of phenolic compounds, which may be related to its high content of isoflavones such as puerarin, daidzein, daidzin, and genistein [13,19].

Partial substitution of wheat flour with kudzu root powder resulted in a statistically significant (*p* ≤ 0.05), substitution-level-dependent increase in both TPC and TFC in the resulting breads. At the highest level of addition (12%, *w*/*w*), the TPC was approximately 24-fold higher compared with the control sample, while TFC increased from an undetectable level to nearly 2.1 mg QE/g d.m. A similar dose–response relationship has been reported in studies on wheat bread enriched with chickpea flour [50], Moringa oleifera leaf powder [51], and quinoa flour [52].

The antioxidant capacity of kudzu root powder, determined using the DPPH and ABTS assays, was approximately 24- and 22-fold higher, respectively, than that of wheat flour. In bread samples, a progressive increase in free radical scavenging activity was observed with increasing levels of kudzu root powder addition. The highest values were recorded for sample BKRF12. In all analyzed samples, the values obtained using the ABTS method were higher than those obtained using the DPPH assay, which is consistent with the analytical characteristics of these methods, as the ABTS radical reacts with both hydrophilic and lipophilic antioxidants [53].

The considerable retention of flavonoid content and antioxidant activity after the baking process may be partially explained by the specific chemical structure of puerarin, the dominant isoflavone present in kudzu root. As a C-glycoside, this compound contains a carbon-carbon bond between the sugar moiety and the aglycone, which exhibits greater resistance to thermal hydrolysis than the O-glycosidic bonds typical of many flavonoids [17]. It also cannot be excluded that the increase in the antioxidant capacity measured in vitro of enriched breads was influenced by Maillard reaction products formed during the baking process.

However, it should be noted that the Folin–Ciocalteu method is not fully specific for phenolic compounds and may also detect other reducing substances present in the analyzed samples. In addition, DPPH and ABTS assays reflect chemical antioxidant capacity measured in vitro and do not directly correspond to physiological antioxidant effects in vivo. Therefore, the obtained results should be interpreted as indicators of antioxidant potential rather than direct biological activity.

### 3.9. Digestible Starch Content

**Figure 4:**
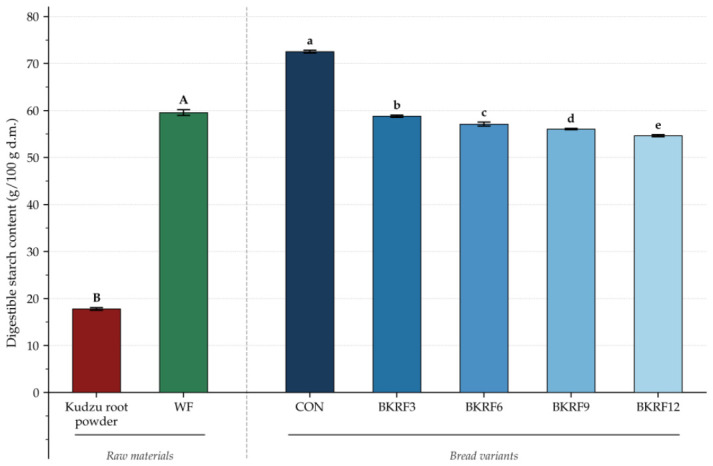
Digestible starch content (g/100 g d.m.) of raw materials and bread with partial substitution of wheat flour with kudzu root powder. Different uppercase letters (A, B) indicate significant differences between raw materials, and lowercase letters (a–e) indicate significant differences among bread samples (Tukey’s test, p ≤ 0.05). Error bars represent standard deviation (n = 3).

The digestible starch content of the raw materials and bread samples is presented in Figure 4. Kudzu root exhibited more than threefold lower digestible starch content compared with wheat flour (*p* ≤ 0.05), which may be related to the overall lower starch content of *Pueraria lobata* root. Unlike refined wheat flour, which consists predominantly of highly gelatinisable starch, kudzu root contains considerable amounts of non-starch components, including dietary fiber, isoflavones and protein [19,54]. In the bread samples, the incorporation of kudzu root powder resulted in a significant and progressive decrease in digestible starch content; at the highest substitution level (12%, *w*/*w*), an approximately 25% reduction relative to the control was observed. All enriched samples differed significantly from both the control and from each other, indicating a clear dose-dependent effect.

The reduction in digestible starch content may be explained by two principal mechanisms. First, the dilution of wheat starch by kudzu root powder, which contains substantially lower amounts of digestible starch. Second, the presence of phenolic compounds is introduced by the kudzu root powder. Isoflavones such as puerarin and daidzein may limit starch digestibility through interactions with starch granules and partial inhibition of α-amylase activity. Polyphenol-starch complexes formed through non-covalent interactions (hydrogen bonding and hydrophobic interactions) may restrict enzyme access to the starch substrate, thereby reducing the rate of starch hydrolysis [55]. In addition, phenolic compounds may contribute to reduced starch hydrolysis, possibly through interactions with α-amylase, with isoflavones demonstrating particularly strong inhibitory potential [56].

From a nutritional perspective, the observed reduction in digestible starch may indicate lower starch availability for enzymatic hydrolysis. The present results indicate that substitution of wheat flour with kudzu root powder influences the digestible starch fraction in bread in a dose-dependent manner. Together with the increased phenolic content and antioxidant capacity discussed previously, this suggests that *P. lobata* root powder may be considered as an ingredient for bakery products with modified nutritional characteristics. However, confirmation of its effect on glycemic response requires further investigation, particularly through glycemic index determination.

## 4. Conclusions

The results of this study demonstrate that ground kudzu root can be successfully incorporated into wheat bread formulations as a functional ingredient rich in dietary fiber. Its addition influenced dough rheology, bread structure, and textural properties, with the magnitude of these changes depending on the substitution level. From a technological perspective, kudzu root modified dough mixing characteristics, increasing apparent dough stability and Farinograph Quality Number (FQN), while slightly increasing water absorption. These changes were most likely associated with enhanced water binding and increased dough viscosity rather than with a direct improvement in gluten strength. At the same time, higher levels of substitution led to increased dough softening, indicating partial weakening of the gluten network. These effects were reflected in bread characteristics, where increased water retention and yield were observed, but excessive substitution (12%) resulted in reduced specific volume and a denser crumb structure. The modification of crumb porosity toward a finer and more uniform structure was accompanied by increased hardness and chewiness, particularly at higher substitution levels. However, breads containing up to 9% kudzu root maintained a favorable balance between technological quality and functional enhancement, with only moderate changes in texture and structure. Enrichment with kudzu root also significantly increased the content of phenolic compounds and antioxidant capacity of the bread, while reducing digestible starch content. Overall, kudzu root shows considerable potential as a novel plant-based ingredient for the development of fiber-enriched bakery products. Future studies should focus on optimizing formulation and processing conditions, as well as evaluating sensory acceptance and the nutritional functionality of the final products.

## Figures and Tables

**Table 1:** Farinograph parameters of wheat dough with partial wheat flour substitution by kudzu root powder (0–12%).

Sample	WA [%]	DDT [min]	ST [min]	DS [FU]	FQN
DCON	59 ± 3 ^a^	5.9 ± 0.3 ^a^	5.9 ± 0.5 ^d^	47 ± 2 ^e^	97 ± 7 ^c^
DKRF3	61 ± 3 ^a^	6.0 ± 0.3 ^a^	6.5 ± 0.4 ^dc^	60 ± 3 ^d^	99 ± 6 ^c^
DKRF6	61 ± 3 ^a^	6.1 ± 0.3 ^a^	7.2 ± 0.4 ^cb^	76 ± 4 ^c^	105 ± 5 ^cb^
DKRF9	62 ± 3 ^a^	6.2 ± 0.3 ^a^	8.0 ± 0.3 ^b^	93 ± 5 ^b^	116 ± 5 ^b^
DKRF12	63 ± 3 ^a^	6.5 ± 0.3 ^a^	10.8 ± 0.3 ^a^	105 ± 5 ^a^	132 ± 5 ^a^

DCON—control dough sample; DKRF—dough with kudzu root powder (3–12, the numbers indicate the percentage of wheat flour substituted with kudzu root); mean value (*n* = 3) ± SD; means in the same column with different letters (a–e) are significantly different (Tukey’s test, *p* ≤ 0.05); WA—water absorption; DDT—dough development time; ST—stability time; DS—dough softening; FQN—Farinograph Quality Number.

**Table 2:** Physical properties of wheat bread with partial wheat flour substitution by kudzu root powder (0–12%).

Sample	Bread Yield [%]	Baking Loss [%]	Specific Volume [cm^3^ g^−1^]	Crumb Moisture after 24 h [%]	Crumb Moisture after 72 h [%]
CON	129.5 ± 0.6 ^c^	10.8 ± 0.4 ^ab^	2.52 ± 0.12 ^a^	44.6 ± 0.1 ^aA^	43.7 ± 0.5 ^aB^
BKRF3	144.0 ± 0.5 ^c^	11.6 ± 0.3 ^a^	2.57 ± 0.11 ^a^	44.5 ± 0.1 ^aA^	44.3 ± 0.7 ^aA^
BKRF6	146.8 ± 0.4 ^b^	10.5 ± 0.3 ^b^	2.53 ± 0.04 ^a^	44.6 ± 0.2 ^aA^	44.2 ± 0.2 ^aA^
BKRF9	148.5 ± 0.5 ^a^	10.9 ± 0.3 ^ab^	2.39 ± 0.03 ^ab^	44.6 ± 0.2 ^aA^	44.3 ± 0.6 ^aA^
BKRF12	148.8 ± 0.6 ^a^	10.5 ± 0.4 ^b^	2.22 ± 0.07 ^b^	44.7 ± 0.2 ^aA^	44.3 ± 0.9 ^aA^

CON—control sample; BKRF—bread with kudzu root powder (3–12, the numbers indicate the percentage of wheat flour substituted with kudzu root); mean value (*n* = 6) ± SD; means in the same column with different letters (a–c) and within a row with different uppercase letters (A, B) are significantly different (Tukey’s test, *p* ≤ 0.05).

**Table 3:** Texture profile analysis of the wheat bread with partial wheat flour substitution by kudzu root powder (0–12%).

Sample	Hardness [N] 24 h	Hardness [N] 72 h	Cohesiveness [-] 24 h	Cohesiveness [-] 72 h	Chewiness [N] 24 h	Chewiness [N] 72 h	Springiness [-] 24 h	Springiness [-] 72 h
CON	9.6 ± 1.2 ^bcA^	14 ± 3 ^aA^	0.58 ± 0.02 ^aA^	0.48 ± 0.06 ^aB^	62 ± 4 ^bA^	61 ± 8 ^bA^	0.93 ± 0.01 ^baA^	0.88 ± 0.02 ^aB^
BKRF3	9.0 ± 1.4 ^bcB^	13 ± 2 ^aA^	0.54 ± 0.06 ^abA^	0.49 ± 0.04 ^aA^	49 ± 6 ^cB^	61 ± 6 ^bA^	0.93 ± 0.01 ^aA^	0.87 ± 0.02 ^aB^
BKRF6	9.7 ± 0.9 ^bcB^	15 ± 2 ^aA^	0.54 ± 0.01 ^abA^	0.42 ± 0.01 ^aB^	55 ± 4 ^bcA^	62 ± 5 ^bA^	0.92 ± 0.01 ^abA^	0.86 ± 0.01 ^aB^
BKRF9	11.3 ± 1.2 ^bB^	14 ± 1 ^aA^	0.52 ± 0.03 ^bA^	0.45 ± 0.13 ^aA^	59 ± 7 ^bcA^	65 ± 4 ^bA^	0.91 ± 0.01 ^bA^	0.85 ± 0.03 ^aB^
BKRF12	15.5 ± 1.0 ^aA^	16 ± 2 ^aA^	0.53 ± 0.05 ^bA^	0.47 ± 0.04 ^aA^	79 ± 4 ^aA^	81 ± 5 ^aA^	0.88 ± 0.01 ^cA^	0.85 ± 0.02 ^aB^

CON—control sample; BKRF—bread with kudzu root powder (3–12, the numbers indicate the percentage of wheat flour substituted with kudzu root); mean value (*n* = 8) ± SD; different lowercase letters (a–c) within a column and different uppercase letters (A, B) within a row indicate statistically significant differences (Tukey’s test, *p* ≤ 0.05).

**Table 4:** Crumb color of wheat bread with partial wheat flour substitution by kudzu root powder (0–12%).

Sample	L*	a*	b*	ΔE*	WI	BI	YI
CON	55 ± 3 ^a^	0.6 ± 0.2 ^e^	13.2 ± 0.6 ^c^	—	53 ± 3 ^a^	1.7 ± 0.4 ^e^	35 ± 2 ^e^
BKRF3	55 ± 3 ^a^	1.7 ± 0.4 ^d^	16.0 ± 1.3 ^b^	4 ± 1 ^c^	52 ± 3 ^a^	3.4 ± 0.7 ^d^	42 ± 5 ^d^
BKRF6	53 ± 3 ^ab^	3.1 ± 0.6 ^c^	18.3 ± 1.6 ^a^	7 ± 2 ^b^	50 ± 3 ^a^	5.5 ± 1.0 ^c^	49 ± 4 ^c^
BKRF9	50 ± 2 ^b^	3.7 ± 0.4 ^b^	18.8 ± 0.8 ^a^	8 ± 1 ^b^	46 ± 2 ^b^	6.8 ± 0.8 ^b^	54 ± 3 ^b^
BKRF12	44 ± 3 ^c^	4.4 ± 0.3 ^a^	18.3 ± 0.7 ^a^	12 ± 2 ^a^	41 ± 2 ^c^	8.7 ± 0.8 ^a^	59 ± 4 ^a^

CON—control sample; BKRF—bread with kudzu root powder (3–12, the numbers indicate the percentage of wheat flour substituted with kudzu root); mean value (*n* = 9) ± SD; means in the same column with different letters (a–e) are significantly different (Tukey’s test, *p* ≤ 0.05); ΔE*—total color difference; WI—whiteness index; BI—browning index; YI—yellowness index.

**Table 5:** Sensory evaluation of wheat bread with partial wheat flour substitution by kudzu root powder (0–12%).

Sample	Appearance	Color	Elasticity and Porosity	Smell	Taste	Overall Acceptability
CON	4.5 ± 0.8 ^a^	4.5 ± 0.9 ^a^	4.1 ± 1.0 ^a^	4.5 ± 0.8 ^a^	4.2 ± 0.9 ^a^	4.4 ± 0.7 ^a^
BKRF3	4.3 ± 0.9 ^ab^	4.1 ± 0.9 ^ab^	4.1 ± 0.9 ^a^	3.8 ± 1.1 ^ab^	3.7 ± 1.2 ^ba^	4.0 ± 0.9 ^ab^
BKRF6	4.1 ± 0.9 ^ab^	3.7 ± 0.9 ^ab^	3.5 ± 1.3 ^a^	3.5 ± 1.3 ^ab^	3.2 ± 1.4 ^ab^	3.6 ± 1.0 ^ab^
BKRF9	3.5 ± 1.2 ^ab^	3.3 ± 1.3 ^b^	3.7 ± 1.3 ^a^	3.4 ± 1.4 ^ab^	2.6 ± 1.2 ^cb^	3.3 ± 1.2 ^ab^
BKRF12	3.3 ± 1.2 ^b^	3.2 ± 1.3 ^b^	3.3 ± 1.2 ^a^	3.1 ± 1.4 ^b^	1.8 ± 1.0 ^c^	2.9 ± 1.1 ^b^

CON—control sample; BKRF—bread with kudzu root (3–12, the numbers indicate the percentage of wheat flour substituted with kudzu root); mean value (*n* = 12) ± SD; means in the same column with different letters (a–c) are significantly different (Tukey’s test, *p* ≤ 0.05).

**Table 6:** Chemical composition of raw materials: wheat flour (WF), kudzu root, and bread with progressive addition of kudzu root powder (0–12%).

Sample	Ash [% d.b.]	Protein [% d.b.]	Fat [% d.b.]	TDF [% d.b.]	IDF [% d.b.]	SDF [% d.b.]	CHO [% d.b.]	Energy [kcal/100 g]
Raw materials								
Kudzu root	5.5 ± 0.2 ^A^	6.6 ± 0.2 ^B^	0.81 ± 0.03 ^A^	41.7 ± 1.1 ^A^	36.6 ± 0.9 ^A^	5.02 ± 0.15 ^A^	45.4 ± 1.4 ^B^	278.2 ± 2.4 ^B^
WF	0.73 ± 0.02 ^B^	13.4 ± 0.4 ^A^	0.45 ± 0.02 ^B^	5.3 ± 0.1 ^B^	2.4 ± 0.1 ^B^	2.9 ± 0.07 ^B^	80.2 ± 0.6 ^A^	340.2 ± 0.2 ^A^
Bread								
CON	2.51 ± 0.05 ^c^	13.8 ± 0.4 ^a^	0.44 ± 0.01 ^b^	6.2 ± 0.2 ^e^	2.5 ± 0.1 ^e^	3.7 ± 0.1 ^a^	77.1 ± 0.7 ^a^	210.4 ± 0.3 ^a^
BKRF3	2.55 ± 0.06 ^c^	13.3 ± 0.2 ^ab^	0.48 ± 0.01 ^ab^	7.1 ± 0.2 ^d^	3.6 ± 0.1 ^d^	3.5 ± 0.1 ^a^	76.6 ± 0.5 ^a^	209.7 ± 0.3 ^a^
BKRF6	2.81 ± 0.08 ^b^	13.1 ± 0.4 ^ab^	0.49 ± 0.01 ^a^	8.1 ± 0.2 ^c^	4.5 ± 0.1 ^c^	3.6 ± 0.1 ^a^	75.5 ± 0.7 ^ab^	207.7 ± 0.4 ^b^
BKRF9	2.91 ± 0.07 ^ab^	12.9 ± 0.3 ^ab^	0.50 ± 0.01 ^a^	9.2 ± 0.2 ^b^	5.4 ± 0.1 ^b^	3.8 ± 0.1 ^a^	74.5 ± 0.6 ^bc^	206.3 ± 0.4 ^c^
BKRF12	3.05 ± 0.07 ^a^	12.6 ± 0.3 ^b^	0.50 ± 0.01 ^a^	10.3 ± 0.3 ^a^	6.7 ± 0.2 ^a^	3.6 ± 0.1 ^a^	73.6 ± 0.7 ^c^	204.5 ± 0.5 ^d^

CON—control sample; WF—wheat flour; BKRF—bread with kudzu root powder (3–12, the numbers indicate the percentage of wheat flour substituted with kudzu root); CHO—available carbohydrate; TDF—total dietary fiber; SDF—soluble dietary fiber; IDF—insoluble dietary fiber; mean value (*n* = 3) ± SD; means in the same column with different lowercase letters (a–e) are significantly different among bread samples, while means with different uppercase letters (A, B) are significantly different among raw materials (Tukey’s test, *p* ≤ 0.05).

**Table 7:** Total phenolic content, total flavonoid content, and antioxidant capacity of raw materials and bread samples with partial substitution of wheat flour with kudzu root powder.

Sample	TPC (mg GAE/g d.m.)	TFC (mg QE/g d.m.)	AC DPPH (mg TE/g d.m.)	AC ABTS (mg TE/g d.m.)
Raw materials				
Kudzu root	40.03 ± 1.21 ^A^	17.21 ± 0.32 ^A^	25.13 ± 1.34 ^A^	32.49 ± 1.43 ^A^
WF	0.18 ± 0.02 ^B^	ND	1.06 ± 0.11 ^B^	1.48 ± 0.18 ^B^
Bread				
CON	0.21 ± 0.03 ^e^	ND	1.13 ± 0.11 ^a^	1.19 ± 0.14 ^e^
BKRF3	1.53 ± 0.12 ^d^	0.56 ± 0.09 ^d^	1.82 ± 0.18 ^b^	1.98 ± 0.31 ^d^
BKRF6	2.78 ± 0.17 ^c^	1.05 ± 0.13 ^c^	2.37 ± 0.09 ^c^	2.64 ± 0.25 ^c^
BKRF9	3.94 ± 0.31 ^b^	1.61 ± 0.12 ^b^	2.82 ± 0.13 ^d^	3.14 ± 0.14 ^b^
BKRF12	5.13 ± 0.38 ^a^	2.12 ± 0.19 ^a^	3.21 ± 0.26 ^e^	3.93 ± 0.31 ^a^

Results are shown as mean ± standard deviation (*n* = 3). Different lowercase letters (a–e) within a column indicate significant differences among bread samples, and different uppercase letters (A, B) indicate significant differences between raw materials according to Tukey’s test (*p* ≤ 0.05). GAE, gallic acid equivalent; QE, quercetin equivalent; TE, Trolox equivalent; d.m., dry matter; ND, not detected; WF, wheat flour; CON, control bread; BKRF3–BKRF12, bread with 3–12% (*w*/*w*) kudzu root powder substitution; AC, antioxidant capacity.

## Data Availability

The original contributions presented in this study are included in the article. Further inquiries can be directed to the corresponding authors.
